# Difference in excess late mortality between early-onset and late-onset cancer survivors: a nationwide cross-sectional study

**DOI:** 10.1097/JS9.0000000000002681

**Published:** 2025-06-20

**Authors:** Jian Li, Xiaohong Kuang

**Affiliations:** a Department of General Surgery, The Third Hospital of Mianyang, Sichuan Mental Health Center, Mianyang, China; b Department of Hematology, The Third Hospital of Mianyang, Sichuan Mental Health Center, Mianyang, China

**Keywords:** early-onset cancer, late mortality, late-onset cancer, public health, socioeconomic deprivation, survivorship

## Abstract

**Background:**

While excess late mortality in early-onset cancer survivors has been documented, no prior study has directly compared these outcomes between early-onset and late-onset survivors.

**Materials and methods:**

This study utilized data collected by the National Health and Nutrition Examination Survey (NHANES) from 1999 to 2018. Participants were adults aged 20 years or older with a history of cancer who survived at least 5 years post-diagnosis. Participants were categorized into early-onset cancer survivors (diagnosed between ages 20 and 49) and late-onset cancer survivors (diagnosed at age 50 or older). Standardized mortality ratios (SMRs) and 95% confidence intervals (CIs) were calculated using age-specific, sex-specific, and calendar year-specific mortality rates from the general population as reference. Incidence rate ratios (IRRs) were estimated using multivariate Poisson regression.

**Results:**

A total of 3082 cancer survivors were included, comprising 1199 (48.3%) early-onset cancer survivors and 1883 (51.7%) late-onset cancer survivors. The overall SMRs were 2.07 (95% CI: 1.76–2.44) for early-onset cancer survivors and 1.59 (95% CI: 1.37–1.84) for late-onset cancer survivors. Compared to late-onset cancer survivors, early-onset cancer survivors exhibited higher late mortality relative to the general population, with an IRR for all-cause mortality of 1.49 (95% CI: 1.19–1.86). This difference was largely associated with malignant neoplasms (IRR: 1.63; 95% CI: 1.12–2.39) rather than other causes of death (IRR: 1.23; 95% CI: 0.95–1.60). When stratified by sociodemographic characteristics, the differences in excess late mortality varied across subgroups.

**Conclusions:**

Our findings suggest that early-onset cancer survivors face higher late mortality relative to the general population compared to late-onset cancer survivors, driven by malignant neoplasms, with disparities exacerbated in socioeconomically disadvantaged groups.

## Introduction

Since the 1990s, an increasing trend in cancer incidence has been observed among populations younger than 50 years^[1-4]^. Given their longer life expectancy, significant efforts have been devoted to investigating cancers affecting this younger demographic, commonly referred to as early-onset cancer. Recognizing its importance, the US National Cancer Institute designated early-onset cancer as a research priority in its 2020–2021 “Provocative Questions” initiative^[5,6]^. This focus holds substantial clinical and societal implications, as distinct tumor characteristics have been identified between early-onset and late-onset cancers (those occurring in patients aged 50 years or older). Compared to late-onset cancers, early-onset cancers are more frequently diagnosed at advanced stages and are associated with higher mortality rates from primary neoplasms^[7-9]^.

Due to their better performance status and lower rates of comorbidities, patients with early-onset cancer often receive more intensive treatments, which may contribute to a higher risk of late mortality among long-term survivors compared to the general population^[10]^. Consequently, despite significant improvements in 5-year survival rates – commonly regarded as an indicator of cancer cure – the excess mortality among long-term survivors has remained largely unchanged^[11-13]^. Several large population-based studies have highlighted that early-onset cancer survivors (primarily adolescents and young adults) face an elevated risk of second primary neoplasms and other chronic health conditions^[5,10,14,15]^. However, these studies have primarily focused on comparing excess mortality in early-onset cancer survivors to the general population, leaving unexplored whether differences in late mortality exist between early-onset and late-onset cancer patients. Critically, early-onset cancer survivors represent a uniquely vulnerable population. As individuals typically diagnosed during their prime working years (20-49 years), they constitute a key demographic for societal economic productivity. Their prolonged life expectancy amplifies both the cumulative burden of treatment-related complications and the population-level impact of excess mortality. Therefore, understanding disparities in late mortality between early- and late-onset cancer survivors is crucial, particularly in light of the rising incidence of early-onset cancer, as it could inform more equitable resource allocation and targeted interventions, not only for clinical care but also for preserving socioeconomic stability.

To address this knowledge gap, we utilized data from the National Health and Nutrition Examination Survey (NHANES) to investigate differences in excess late mortality between early-onset and late-onset cancer patients.

## Methods

### Data collection

Data for this analysis were obtained from NHANES, a nationally representative serial cross-sectional study initiated in 1999. NHANES employs a complex multistage probabilistic cluster sampling design to biennially survey the civilian, non-institutionalized US population, with the primary aim of assessing the health and nutritional status of the population. The study was approved by the National Center for Health Statistics Ethics Review Board, and detailed descriptions of the study design and informed consent procedures are available online^[16]^. For this study, participants from 10 survey cycles (1999–2018) who met the following criteria were included: (1) adults aged 20 years or older; (2) a reported history of cancer, including age at diagnosis; (3) survival for at least 5 years post-diagnosis, a threshold generally considered indicative of cancer cure (Supplemental Digital Content, Figure S1, available at: http://links.lww.com/JS9/E442). Data on cancer diagnosis and sociodemographic characteristics were extracted for analysis.

As a secondary analysis of publicly available data that was anonymized, informed consent and institutional review board approval were not required. This study was conducted according to the Strengthening the Reporting of Cohort, Cross-sectional and Case-control Studies in Surgery 2025 (STROCSS 2025) guidelines^[17]^.

### Definition of cancer survivors

During household interviews, participants were asked to report their cancer history, including cancer type and age at diagnosis. Based on this information, participants were categorized into two groups: early-onset cancer survivors (diagnosed between ages 20 and 49); late-onset cancer survivors (diagnosed at age 50 or older). While the use of arbitrary age cutoffs has limitations and heterogeneity exists among cancers within the same age range, these definitions were selected for consistency, simplicity, and data comparability^[6]^. Due to the limited number of cases for certain cancer types, cancers were categorized into the following groups: breast cancer, female genital cancer (ovarian, cervical, and uterine), melanoma, skin cancer, gastrointestinal cancer (colon, esophageal, gallbladder, liver, pancreatic, rectal, stomach), hematological cancer (blood, leukemia, lymphoma), and others (brain, bone, nervous system, head and neck cancers that have insufficient cases for individual analysis) (Supplemental Digital Content, Table S1, available at: http://links.lww.com/JS9/E442).

**HIGHLIGHTS**

- An increased risk for late mortality was observed in long-term survivors from early-onset cancer, whereas the potential difference in excess late mortality between early-onset and late-onset cancer survivors remains unexplored.
- Early-onset cancer survivors experience higher late mortality relative to the general population compared to late-onset cancer survivors.
- The elevated risk of excess late mortality among early-onset cancer survivors is primarily driven by malignant neoplasms.
- Disparities in excess late mortality are more pronounced among women, non-Hispanic White individuals, and those with lower socioeconomic status.

### Assessment of sociodemographic characteristics

Sociodemographic characteristics, including sex, race, education level, and family economic status, were included due to their potential associations with cancer survival. Race and ethnicity were collected through standardized questionnaires and categorized as non-Hispanic White, non-Hispanic Black, Hispanic (including Mexican American and other Hispanic), and other races (including non-Hispanic Asian). Education level was classified as less than high school, high school, and college or above, consistent with previous NHANES studies^[18,19]^. Family economic status was assessed using the family income-to-poverty ratio, categorized as <1.30 (low income), 1.30–3.49 (middle income), and ≥3.50 (high income) (Supplemental Digital Content, Table S1, available at: http://links.lww.com/JS9/E442)^[18,19]^.

### Mortality determination and follow-up

Mortality data for the study population were obtained through linkage to the National Death Index (NDI) records. The detailed methodology for this linkage has been described elsewhere^[20]^. Participants were passively followed from the date of the household interview until death or 31 December 2019, the latest update date for the Linked Mortality Files, which are publicly available for download. Mortality was categorized based on the underlying cause of death into all-cause mortality, cancer-related mortality, and non-cancer-related mortality. Non-cancer-related mortality included deaths caused by heart disease, chronic lower respiratory disease, accidents, cerebrovascular diseases, Alzheimer’s disease, diabetes, influenza and pneumonia, nephritis, nephrotic syndrome, nephrosis, and other causes.

### Statistical analysis

In accordance with NHANES analytical guidelines, survey analysis procedures were incorporated into all analyses to account for sample weights, stratification, and clustering, reflecting the complex sampling design^[21]^.

Descriptive statistics were generated for sociodemographic characteristics, primary cancer types, and underlying causes of death for early-onset and late-onset cancer survivors. Differences between the two groups were assessed using t-tests and chi-square tests. Given that the mean age of early-onset cancer survivors was younger and the background mortality rate in older populations is significantly higher, direct comparison of mortality rates between the two groups was not feasible. Therefore, standardized mortality ratios (SMRs) and 95% confidence intervals (CIs) were calculated for early-onset and late-onset cancer survivors using age-specific, sex-specific, and calendar year-specific mortality rates from the general population, as provided by the Centers for Disease Control and Prevention’s Wide-Ranging Online Data for Epidemiologic Research (CDC WONDER) database.

Incidence rate ratios (IRRs), defined as the ratio of the SMR in early-onset cancer to that in late-onset cancer, were estimated using multivariate Poisson regression for all-cause, cancer-related, and non-cancer-related mortality. As SMR has already accounted for sex, age, and calendar year, covariates in the regression models included race and ethnicity, education level, family income-to-poverty ratio, survival time, and cancer type. Subgroup analyses were stratified by sex, race and ethnicity, education level, family income-to-poverty ratio, cancer type, survival time (5–9, 10–14, 15–19, and ≥20 years), age at screening (55–64, 65–74, and ≥75 years), and diagnosis era (1940–1979, 1980–1989, 1990–1999, and 2000–2020).

All statistical analyses were performed using IBM SPSS Statistics 25 (IBM Co., Armonk, NY, USA) and STATA 14.0 (Stata Corporation, Texas, USA). A two-sided P value <0.05 was considered statistically significant. The sample size was determined by the available pool of eligible long-term cancer survivors within the NHANES dataset rather than by formal power calculations. P values for multiple comparisons were not adjusted; therefore, secondary analyses should be interpreted as exploratory.

## Results

### Participant characteristics

A total of 3082 cancer survivors meeting the inclusion criteria were included in the analysis, comprising 1199 (48.3%) early-onset cancer survivors and 1883 (51.7%) late-onset cancer survivors. Early-onset survivors were younger at enrollment (mean 54.6 ± 0.4 years) than late-onset survivors (72.1 ± 0.3 years). Early-onset cancer survivors were more likely to be women, accounting for 69.4% (836 cases), whereas the sex distribution was nearly equal among late-onset cancer survivors, with 1046 (49.1%) men and 837 (50.9%) women. The proportion of non-Hispanic White individuals was lower among early-onset cancer survivors (84.0% vs. 89.4%). Additionally, early-onset cancer survivors had higher rates of college or above education levels (66.3% vs. 54.1%) and high income level (59.9% vs. 44.4%) compared to late-onset survivors. A higher proportion of early-onset cancer survivors were diagnosed before 1979 (19.9% vs. 2.4%), whereas a lower proportion were diagnosed after 2000 (22.9% vs. 41.9%). Skin cancer and melanoma were the most common cancer types in both groups. Female genital cancers were more prevalent among early-onset cancer survivors, accounting for 25.4% of cases compared to only 5.0% in late-onset survivors. Other solid cancers, such as gastrointestinal, breast, and lung cancers, were more frequently observed in late-onset cancer survivors (Supplemental Digital Content, Table S2, available at: http://links.lww.com/JS9/E442).

### Underlying leading causes of death

**Figure 1.**
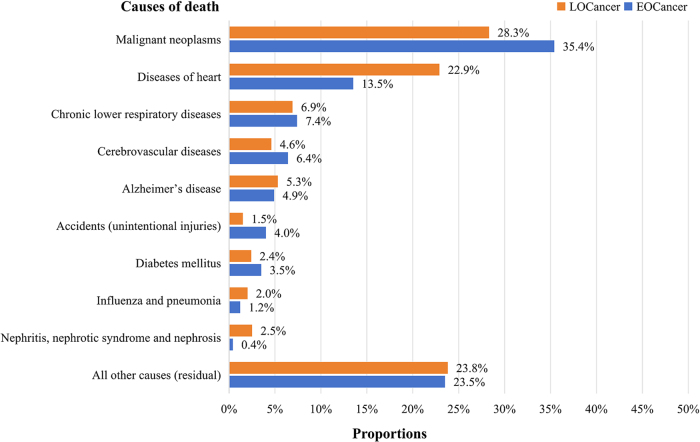
The underlying leading causes of late death in early-onset and late-onset cancer survivors.

Among the 3082 eligible at least 5-year cancer survivors, the median follow-up duration from the interview was 6.9 years (range: 0–20 years). During follow-up, 248 deaths (16.7%) occurred in early-onset survivors and 831 (40.7%) in late-onset survivors. Malignant neoplasms, heart disease, chronic lower respiratory diseases, and cerebrovascular diseases were the leading causes of death in both groups. However, early-onset cancer survivors had a higher proportion of deaths attributed to malignant neoplasms (35.4% vs. 28.3%) and a lower proportion due to heart disease (13.5% vs. 22.9%) compared to late-onset cancer survivors. The weighted proportions of these four leading causes of death were 35.4%, 13.5%, 7.4%, and 6.4% for early-onset cancer survivors and 28.3%, 22.9%, 6.9%, and 4.6% for late-onset cancer survivors, respectively (Fig. 1).

### Difference in all-cause excess late mortality

**Figure 2.**
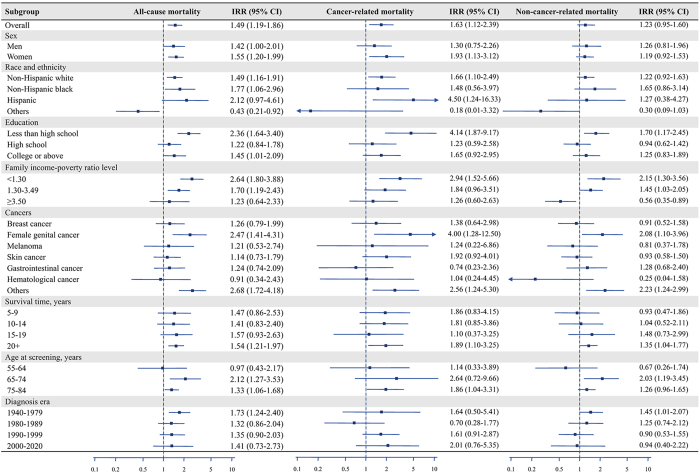
Incidence rate ratios and 95% confidence intervals of early-onset cancer survivors over late-onset cancer survivors. Incidence rate ratios (IRRs) with 95% confidence intervals (CIs) greater than 0 indicate positive associations, whereas those with 95% CIs less than 0 indicate negative associations; otherwise, no significant associations exist.

The all-cause SMRs for early-onset and late-onset cancer survivors were 2.07 (95% CI: 1.76–2.44) and 1.59 (95% CI: 1.37–1.84), respectively. Early-onset cancer survivors exhibited higher excess late mortality relative to the general population compared to late-onset survivors, with an IRR for all-cause mortality of 1.49 (95% CI: 1.19–1.86). When stratified by sociodemographic characteristics, the difference in excess late mortality was most pronounced among women (IRR: 1.55; 95% CI: 1.20–1.99), non-Hispanic White individuals (IRR: 1.49; 95% CI: 1.16–1.91), those with lower education levels (IRR: 2.36; 95% CI: 1.64–3.40), and those with lower income (IRR: 2.64; 95% CI: 1.80–3.88). Significant differences were also observed among survivors of female genital cancer (IRR: 2.47; 95% CI: 1.43–4.31) and other cancers (IRR: 2.68; 95% CI: 1.72–4.81). Additionally, the difference in excess late mortality was more notable among survivors with a survival time exceeding 20 years, those older than 65 years, and those diagnosed before 1979 (Table 1 and Fig. 2).

**Table 1:** Observed all cause deaths and standardized mortality ratios for early-onset and late-onset cancer survivors, 1999–2018

Subgroup	Early-onset cancer, n (weighted %)			Late-onset cancer, n (weighted %)		
	Alive	Dead	SMR (95% CI)	Alive	Dead	SMR (95% CI)
Overall	951 (83.3)	248 (16.7)	2.07 (1.76–2.44)	1052 (59.3)	831 (40.7)	1.59 (1.37–1.84)
Sex						
Men	251 (81.6)	85 (18.4)	1.75 (1.33–2.31)	543 (55.3)	503 (44.7)	1.60 (1.45–1.76)
Women	700 (84.0)	163 (16.0)	2.28 (1.93–2.69)	509 (63.2)	328 (36.8)	1.86 (1.68–2.05)
Race and ethnicity						
Non-Hispanic white	605 (82.9)	181 (17.1)	2.00 (1.66–2.41)	721 (58.7)	671 (41.3)	1.70 (1.58–1.84)
Non-Hispanic black	115 (75.0)	42 (25.0)	3.79 (2.76–5.19)	153 (57.5)	101 (42.5)	2.13 (1.74–2.61)
Hispanic	166 (88.9)	20 (11.1)	2.21 (1.26–3.89)	122 (75.6)	37 (24.4)	1.26 (0.90–1.76)
Others	65 (92.9)	5 (7.1)	1.38 (0.48–3.95)	56 (67.4)	22 (32.6)	1.65 (1.00–2.71)
Education						
Less than high school	160 (67.6)	75 (32.4)	4.05 (3.15–5.23)	195 (38.0)	254 (62.0)	2.11 (1.88–2.36)
High school	196 (80.9)	50 (19.1)	2.27 (1.70–3.02)	230 (50.0)	218 (50.0)	2.04 (1.82–2.30)
College or above	594 (86.9)	123 (13.1)	1.65 (1.29–2.10)	626 (68.9)	359 (31.1)	1.42 (1.27–1.58)
Family income-poverty ratio level						
<1.30	221 (67.9)	81 (32.1)	4.81 (3.76–6.14)	180 (46.3)	181 (53.7)	2.25 (1.96–2.60)
1.30–3.49	267 (78.1)	94 (21.9)	2.43 (1.91–3.10)	405 (53.1)	357 (46.9)	1.73 (1.57–1.91)
≥3.50	389 (90.8)	57 (9.2)	1.17 (0.88–1.57)	362 (69.3)	213 (30.7)	1.47 (1.27–1.71)
Cancers						
Breast cancer	139 (84.7)	40 (15.3)	2.09 (1.41–3.09)	197 (58.4)	141 (41.6)	1.93 (1.68–2.21)
Female genital cancer	310 (83.4)	69 (16.6)	2.82 (2.16–3.67)	47 (69.4)	30 (30.6)	1.69 (1.24–2.31)
Melanoma	71 (89.1)	11 (10.9)	1.35 (0.76–2.40)	67 (73.4)	34 (26.6)	1.17 (0.81–1.70)
Skin cancer	186 (84.3)	51 (15.7)	1.47 (1.06–2.03)	239 (63.7)	179 (36.3)	1.54 (1.30–1.83)
Gastrointestinal cancer	43 (74.2)	16 (25.8)	2.26 (1.32–3.86)	97 (50.4)	102 (19.6)	2.07 (1.67–2.56)
Hematological cancer	34 (84.0)	8 (16.0)	3.24 (1.44–7.27)	22 (15.0)	22 (55.0)	2.83 (1.80–4.44)
Others	168 (79.4)	46 (20.6)	2.07 (1.76–2.44)	367 (54.8)	321 (45.2)	1.70 (1.59–1.84)
Survival time, years						
5–9	241 (90.9)	24 (9.1)	2.47 (1.48–4.15)	490 (61.7)	331 (38.3)	1.82 (1.60–2.08)
10–14	183 (90.3)	24 (9.7)	2.28 (1.41–3.71)	297 (57.5)	258 (42.5)	1.74 (1.54–1.97)
15–19	150 (86.2)	22 (13.8)	2.06 (1.28–3.31)	149 (59.8)	117 (40.2)	1.64 (1.42–1.90)
20+	377 (74.1)	178 (25.9)	1.97 (1.67–2.34)	116 (54.3)	125 (45.7)	1.43 (1.24–1.64)
Age at screening, years						
55–64	266 (83.9)	59 (16.1)	2.07 (1.44–2.98)	185 (74.3)	57 (25.7)	2.75 (1.96–3.83)
65–74	133 (66.6)	68 (33.4)	2.14 (1.70–2.71)	452 (67.5)	227 (32.5)	1.75 (1.54–1.99)
75–84	56 (46.7)	79 (53.3)	1.91 (1.60–2.28)	415 (45.8)	547 (54.2)	1.58 (1.47–1.69)
Diagnosis era						
1940–1979	152 (58.8)	136 (41.2)	2.24 (1.87–2.68)	4 (11.1)	41 (88.9)	1.78 (1.50–2.12)
1980–1989	235 (84.5)	55 (15.5)	1.79 (1.31–2.44)	91 (35.8)	185 (64.2)	1.80 (1.60–2.03)
1990–1999	291 (88.5)	45 (11.5)	2.30 (1.60–3.31)	351 (48.2)	412 (51.8)	1.87 (1.69–2.07)
2000–2020	273 (96.0)	12 (4.0)	1.53 (0.74–3.16)	606 (81.1)	193 (18.9)	1.31 (1.09–1.58)

SMR, standardized mortality ratio; CI, confidence interval.

### Difference in cancer-related excess late mortality

The cancer-related SMRs for early-onset and late-onset cancer survivors were 3.05 (95% CI: 2.30–4.06) and 2.66 (95% CI: 2.27–3.13), respectively. Early-onset cancer survivors exhibited higher cancer-related excess late mortality relative to the general population compared to late-onset survivors, with an IRR for cancer-related mortality of 1.63 (95% CI: 1.12–2.39). Similar to all-cause excess late mortality, the difference in cancer-related excess late mortality was most pronounced among women (IRR: 1.93; 95% CI: 1.13–3.12), non-Hispanic White individuals (IRR: 1.66; 95% CI: 1.10–2.49), those with lower education levels (IRR: 4.14; 95% CI: 1.87–9.17), and those with lower income (IRR: 2.94; 95% CI: 1.52–5.66). Significant differences were also observed among survivors of female genital cancer (IRR: 4.00; 95% CI: 1.28–12.50) and other cancers (IRR: 2.56; 95% CI: 1.24–5.30). Additionally, the difference in cancer-related excess late mortality was more notable among survivors with a survival time exceeding 20 years and those older than 75 years, whereas no significant differences were observed based on diagnosis era (Table 2 and Fig. 2).

**Table 2:** Observed cancer-related deaths and standardized mortality ratios for early-onset and late-onset cancer survivors, 1999–2018

Subgroup	Early-onset cancer, n (weighted %)			Late-onset cancer, n (weighted %)		
	Alive	Dead	SMR (95% CI)	Alive	Dead	SMR (95% CI)
Overall	951 (93.4)	79 (6.6)	3.05 (2.30–4.06)	1052 (83.8)	234 (16.2)	2.66 (2.27–3.13)
Sex						
Men	251 (93.5)	28 (6.5)	2.42 (1.47–3.97)	543 (79.5)	157 (20.5)	2.77 (2.33–3.28)
Women	700 (93.3)	51 (6.7)	3.43 (2.42–4.86)	509 (87.7)	77 (12.3)	2.52 (1.92–3.30)
Race and ethnicity						
Non-Hispanic white	605 (93.0)	58 (7.0)	3.03 (2.19–4.19)	721 (83.4)	182 (16.6)	2.67 (2.25–3.18)
Non-Hispanic black	115 (90.7)	13 (9.3)	5.39 (2.61–11.12)	153 (79.3)	34 (20.7)	3.81 (2.65–5.49)
Hispanic	166 (96.3)	6 (3.7)	2.75 (1.12–6.77)	122 (94.5)	11 (5.5)	1.14 (0.64–2.02)
Others	65 (98.6)	2 (1.4)	1.00 (0.21–4.80)	56 (90.1)	7 (9.9)	1.81 (0.74–4.44)
Education						
Less than high school	160 (85.3)	26 (14.7)	7.12 (4.22–12.03)	195 (74.2)	57 (25.8)	3.46 (2.48–4.82)
High school	196 (90.9)	17 (9.1)	4.08 (2.45–6.79)	230 (77.3)	64 (22.7)	3.55 (2.72–4.63)
College or above	594 (95.4)	36 (4.6)	2.12 (1.35–3.33)	626 (87.6)	113 (12.4)	2.16 (1.76–2.65)
Family income-poverty ratio level						
<1.30	221 (87.5)	27 (12.5)	8.46 (5.06–14.12)	180 (79.7)	51 (20.3)	3.75 (2.70–5.20)
1.30–3.49	267 (91.8)	27 (8.2)	3.48 (2.07–5.85)	405 (80.5)	95 (19.5)	2.90 (2.31–3.65)
≥3.50	389 (95.4)	21 (4.6)	2.08 (1.24–3.49)	362 (86.5)	73 (13.5)	2.34 (1.80–3.04)
Cancers						
Breast cancer	139 (91.3)	19 (8.7)	3.98 (2.19–7.23)	197 (85.4)	37 (14.6)	2.88 (1.98–4.21)
Female genital cancer	310 (94.6)	15 (5.4)	3.44 (1.77–6.68)	47 (88.2)	7 (11.8)	2.58 (1.16–5.72)
Melanoma	71 (95.0)	3 (5.0)	2.25 (0.68–7.42)	67 (90.5)	9 (9.5)	1.55 (0.70–3.44)
Skin cancer	186 (94.9)	15 (5.1)	1.85 (0.93–3.68)	239 (91.0)	29 (9.0)	1.44 (0.87–2.38)
Gastrointestinal cancer	43 (91.5)	5 (8.5)	2.74 (0.96–7.83)	97 (78.4)	27 (21.6)	3.32 (2.14–5.15)
Hematological cancer	34 (88.9)	6 (11.1)	8.62 (3.65–20.41)	22 (51.8)	16 (48.2)	9.06 (5.17–15.89)
Others	168 (91.0)	16 (9.0)	4.00 (2.46–6.52)	383 (78.1)	109 (21.9)	3.22 (2.68–3.87)
Survival time, years						
5–9	241 (95.1)	9 (4.9)	4.91 (2.23–10.82)	490 (82.4)	113 (17.6)	3.08 (2.42–3.92)
10–14	183 (95.1)	12 (4.9)	4.24 (2.05–8.78)	297 (84.5)	64 (15.5)	2.46 (1.85–3.27)
15–19	150 (94.4)	7 (5.6)	2.66 (1.08–6.56)	149 (84.7)	31 (15.3)	2.51 (1.76–3.60)
20+	377 (90.7)	51 (9.3)	2.57 (1.80–3.69)	116 (87.1)	26 (12.9)	1.77 (1.22–2.57)
Age at screening, years						
55–64	266 (92.7)	26 (7.3)	2.72 (1.62–4.55)	185 (88.7)	21 (11.3)	3.51 (2.04–6.05)
65–74	133 (88.0)	16 (12.0)	2.36 (1.40–3.97)	452 (86.4)	79 (13.6)	2.29 (1.76–2.98)
75–84	56 (77.6)	20 (22.4)	3.51 (2.16–5.70)	415 (77.7)	134 (22.3)	2.80 (2.32–3.89)
Diagnosis year						
1940–1979	152 (81.3)	40 (18.7)	3.63 (2.40–5.48)	4 (46.1)	8 (53.9)	4.22 (2.10–8.49)
1980–1989	235 (96.1)	14 (3.9)	1.48 (0.75–2.92)	91 (70.7)	41 (29.3)	3.02 (2.14–4.26)
1990–1999	291 (93.8)	20 (6.2)	4.21 (2.45–7.21)	351 (75.9)	121 (24.1)	3.12 (2.51–3.89)
2000–2020	273 (97.7)	5 (2.3)	3.25 (1.10–9.62)	606 (92.6)	64 (7.4)	1.81 (1.29–2.52)

CI, confidence interval; SMR, standardized mortality ratio.

### Difference in non-cancer-related excess late mortality

The non-cancer-related SMRs for early-onset and late-onset cancer survivors were 2.09 (95% CI: 1.74–2.52) and 1.98 (95% CI: 1.83–2.14), respectively. The overall difference in non-cancer-related excess late mortality between early-onset and late-onset cancer survivors was not significant, with an IRR of 1.23 (95% CI: 0.95–1.60). However, significant differences were observed in certain subgroups, including those with lower education levels (IRR: 1.70; 95% CI: 1.17–2.45), lower income (IRR: 2.15; 95% CI: 1.30–3.56), female genital cancer (IRR: 2.08; 95% CI: 1.10–3.96), and other cancers (IRR: 2.23; 95% CI: 1.24–2.99). Significant differences were also found among survivors with a survival time exceeding 20 years (IRR: 1.35; 95% CI: 1.04–1.77) and those diagnosed before 1979 (IRR: 1.45; 95% CI: 1.01–2.07) (Table 3 and Fig. 2).

**Table 3:** Observed non-cancer-related deaths and standardized mortality ratios for early-onset and late-onset cancer survivors, 1999–2018

Subgroup	Early-onset cancer, n (weighted %)			Late-onset cancer, n (weighted %)		
	Alive	Dead	SMR (95% CI)	Alive	Dead	SMR (95% CI)
Overall	951 (88.5)	169 (11.5)	2.09 (1.74–2.52)	1052 (67.1)	597 (32.9)	1.98 (1.83–2.14)
Sex						
Men	251 (86.5)	57 (13.5)	1.81 (1.30–2.53)	543 (64.5)	346 (35.5)	1.80 (1.59–2.03)
Women	700 (89.4)	112 (10.6)	2.29 (1.91–2.76)	509 (69.4)	251 (30.6)	2.21 (2.00–2.45)
Race and ethnicity						
Non-Hispanic white	605 (88.4)	123 (11.6)	1.99 (1.63–2.42)	721 (66.4)	489 (33.6)	1.97 (1.81–2.14)
Non-Hispanic black	115 (81.3)	29 (18.7)	4.06 (2.86–5.76)	153 (67.6)	67 (32.4)	2.31 (1.78–2.99)
Hispanic	166 (92.0)	14 (8.0)	2.57 (1.24–5.32)	122 (79.1)	26 (20.9)	1.61 (1.04–2.50)
Others	65 (94.2)	3 (5.8)	1.70 (0.47–6.14)	56 (72.9)	15 (27.1)	2.14 (1.19–3.83)
Education						
Less than high school	160 (76.6)	49 (23.4)	4.38 (3.34–5.75)	195 (43.8)	197 (56.2)	2.62 (2.31–2.98)
High school	196 (88.0)	33 (12.0)	2.06 (1.44–2.94)	230 (58.6)	154 (41.4)	2.31 (1.78–2.99)
College or above	160 (76.6)	49 (23.4)	1.73 (1.34–2.24)	195 (43.8)	197 (56.2)	1.61 (1.04–2.50)
Family income-poverty ratio level						
<1.30	221 (75.2)	54 (24.8)	5.47 (4.04–7.42)	180 (52.5)	130 (47.5)	2.82 (2.37–3.37)
1.30–3.49	267 (84.0)	67 (16.0)	2.66 (2.09–3.38)	405 (60.9)	262 (39.1)	2.00 (1.80–2.23)
≥3.50	389 (94.9)	36 (5.1)	0.94 (0.68–1.30)	362 (77.8)	140 (22.2)	1.58 (1.31–1.90)
Cancers						
Breast cancer	139 (92.1)	21 (7.9)	1.71 (1.00–2.94)	197 (64.9)	104 (35.1)	2.35 (2.01–2.74)
Female genital cancer	310 (87.5)	54 (12.5)	3.25 (2.48–4.25)	47 (76.5)	23 (23.5)	1.92 (1.36–2.71)
Melanoma	71 (93.5)	8 (6.5)	1.17 (0.58–2.39)	67 (79.5)	25 (20.5)	1.33 (0.86–2.05)
Skin cancer	186 (88.3)	36 (11.7)	1.56 (1.12–2.16)	239 (68.0)	150 (32.0)	1.94 (1.64–2.29)
Gastrointestinal cancer	43 (79.8)	11 (20.2)	2.55 (1.34–4.84)	97 (58.5)	75 (41.5)	2.45 (1.88–3.19)
Hematological cancer	34 (93.9)	2 (6.1)	1.80 (0.34–9.54)	22 (77.5)	6 (22.5)	1.32 (0.73–2.37)
Others	168 (86.2)	37 (13.8)	2.47 (1.55–3.93)	383 (64.8)	214 (35.2)	1.86 (1.62–2.14)
Survival time, years						
5–9	241 (95.4)	15 (4.6)	1.71 (0.88–3.33)	490 (71.0)	218 (29.0)	2.01 (1.71–2.36)
10–14	183 (94.7)	12 (5.3)	1.73 (0.92–3.26)	297 (64.3)	194 (35.7)	2.09 (1.83–2.40)
15–19	150 (90.8)	15 (9.2)	2.08 (1.17–3.69)	149 (67.1)	86 (32.9)	1.89 (1.57–2.27)
20+	377 (80.3)	127 (19.7)	2.23 (1.86–2.68)	116 (59.0)	99 (41.0)	1.79 (1.51–2.12)
Age at screening, years						
55–64	266 (89.9)	33 (10.1)	1.98 (1.25–3.12)	185 (82.1)	36 (17.9)	2.96 (1.90–4.61)
65–74	133 (73.3)	52 (26.7)	2.61 (1.93–3.52)	452 (75.6)	148 (24.4)	1.99 (1.71–2.32)
75–84	56 (54.0)	59 (46.0)	2.21 (1.81–2.70)	415 (52.7)	413 (47.3)	1.87 (1.72–2.03)
Diagnosis year						
1940–1979	152 (68.0)	96 (32.0)	2.45 (2.02–2.96)	4 (12.8)	33 (87.2)	2.34 (1.88–2.90)
1980–1989	235 (87.6)	41 (12.4)	2.17 (1.51–3.12)	91 (42.0)	144 (58.0)	2.23 (1.97–2.51)
1990–1999	291 (94.0)	25 (6.0)	1.68 (1.02–2.78)	351 (57.0)	291 (43.0)	2.19 (1.94–2.47)
2000–2020	273 (98.2)	7 (1.8)	0.95 (0.39–2.31)	606 (86.8)	128 (13.2)	1.33 (1.07–1.67)

CI, confidence interval; SMR, standardized mortality ratio.

## Discussion

### Principal findings

To our knowledge, this is among the first studies to investigate the difference in excess late mortality between early-onset and late-onset cancer survivors. Given that early-onset cancer survivors are younger than their late-onset counterparts and that background mortality rates are inherently higher in older populations, direct comparison of survival times between these groups is unreasonable. Therefore, we utilized SMRs and IRRs to quantify these differences. Our findings indicate that early-onset cancer survivors exhibit higher late mortality relative to the general population compared to late-onset cancer survivors. This difference was largely attributed to malignant neoplasms rather than other causes of death. However, the findings varied across different sociodemographic groups. These findings may have public health implications, as they indicate that targeted screening and interventions may reduce late mortality in specific cancer survivor populations.

Compared with previous studies, the excess risk for late mortality was lower in both early-onset and late-onset cancer survivors in our analysis^[5,10,14,15]^. The reasons are multifaceted. First, the cross-sectional design of NHANES relied on retrospective confirmation of cancer survivors, potentially excluding those who died before inclusion and leading to an underestimation of excess late mortality. Second, previous studies primarily focused on survivors of adolescent and young adult cancers, where the mean age of participants was younger, and background mortality rates in the corresponding general population were very low, making excess late mortality more pronounced. Nevertheless, our findings suggest that both early-onset and late-onset cancer survivors have a higher risk of late mortality than the general population, with the risk being more significant in early-onset cancer survivors.

The mechanisms underlying this disparity are not fully understood. Over the past three decades, advancements in therapeutic strategies have greatly improved the 5-year relative survival rates for childhood and late-onset cancers; however, similar improvements have not been achieved for young adult populations, possibly due to lower incidence rates and limited clinical trial enrollment^[11-13]^. Furthermore, studies suggest that early-onset cancers are often diagnosed at later stages and exhibit greater invasiveness compared to late-onset cancers^[6,8,9]^. Although these factors primarily influence 5-year survival rates, recurrence or progression after 5-year survival is common in some early-onset cancers^[5]^. However, as this study could not distinguish between deaths caused by primary or secondary cancers, the extent to which late recurrence or progression contributes to the increased risk of late mortality in early-onset cancer survivors remains unknown. Additionally, the better performance status and lower comorbidity burden in early-onset cancer patients often lead to more intensive treatments. For example, compared with late-onset patients, early-onset gastric cancer patients underwent more extensive D2 lymphadenectomy and neoadjuvant chemotherapy^[22]^. Furthermore, the proportions of patients completed the perioperative therapy were higher in early-onset colorectal cancer^[23]^. In combination with evidence that initial treatment intensity has been identified as a significant risk factor for late mortality in adolescent and young adult cancer survivors^[10]^, these findings indirectly suggest that intensive treatment could contribute to the higher SMRs in early-onset cancer survivors. This hypothesis is supported by our subgroup analyses, which found no significant differences in cancer types associated with lower treatment intensity, such as melanoma, breast cancer, and skin cancer. However, no treatment-related data are available in this study, whether the differences in late mortality associated with treatment intensity needs validation in cohorts with treatment records. This hypothesis may be inconsistent with our findings that the differences were mainly observed in those diagnosed before 1979, long before modern cancer treatments have been established. Such inconsistency also reflects the limitation of our study design. The attenuated mortality disparity in the latest periods may reflect shorter follow-up for younger survivors rather than true therapeutic effects. Prospective studies with treatment data and longer observation are needed to disentangle era effects from age and survivor biases.

In this study, we found that the difference in excess late mortality between early-onset and late-onset cancer survivors was primarily driven by malignant neoplasms rather than other causes of death. On one hand, these findings suggest that late recurrence or progression of primary cancer or the development of secondary cancers may be more common in early-onset cancer survivors. On the other hand, the relatively older age of late-onset cancer survivors increases their risk of death from cardiopulmonary diseases. Therefore, differences in excess late mortality were more pronounced among older survivors. Another interesting finding is that early-onset cancer survivors with lower education and income levels had higher SMRs than their late-onset counterparts. Previous studies have reported that socioeconomic deprivation increases all-cause and cardiovascular disease mortality in the general population^[24]^. The reasons why socioeconomic deprivation has a more significant impact on early-onset cancer survivors remain unclear but may be attributed to differences in cancer characteristics and treatment intensity. Lower socioeconomic status is associated with delayed diagnosis and more advanced tumor stages in younger populations who are not covered by screening. Financial toxicity may further reduce adherence to long-term follow-up and supportive care, exacerbating disparities in late mortality. Nevertheless, our findings suggest that improving healthcare access through social and fiscal policies could mitigate disparities in excess late mortality.

### Implications for public health

The findings of this study underscore the need for targeted public health strategies to address the elevated late mortality risk among early-onset cancer survivors. First, enhanced surveillance programs should be implemented to monitor long-term survivors for late recurrence, secondary cancers, and treatment-related complications. Second, healthcare providers should prioritize personalized follow-up care, particularly for survivors from disadvantaged socioeconomic backgrounds, to address barriers to access and adherence. Third, public health campaigns should focus on raising awareness about the unique challenges faced by early-onset cancer survivors, including the importance of lifestyle modifications and early detection of chronic conditions. Finally, policy interventions, such as expanding insurance coverage and providing financial support for long-term care, could help reduce disparities in excess late mortality. These measures, combined with ongoing research into the biological and social determinants of excess late mortality, could significantly improve outcomes for this vulnerable population.

### Strengths and limitations

A key strength of this study is the use of a nationally representative dataset, which enhances the generalizability of our findings. However, several limitations should be acknowledged. First, cancer diagnoses were self-reported, which may introduce recall bias. Nevertheless, cancer diagnosis is an important event in life, recall bias is unlikely to severely affect the confidence of our findings. Second, the follow-up was relatively short and the time interval between age at diagnosis and the start of follow-up varied among participants, potentially affecting the accuracy of mortality assessments. Third, the limited number of cases precluded detailed analyses of specific cancer types and non-cancer causes of death. Fourth, cancer stage, treatment and comorbidities may be causes for late-mortality disparity. The absence of these data may limit the evidence base to support clinical decision-making and public health policy formulation for cancer survivorship care. Fifth, no formal power calculation and the small sample sizes resulted in limited precision for certain subgroup estimates, which should be interpreted as exploratory. Finally, the cross-sectional design may have excluded many cancer survivors who died before inclusion. This survival bias could make observed disparities between early- and late-onset cohorts inconsistent with the truth. Therefore, our findings need to be validated by prospective studies.

## Conclusion

Our analyses suggest that early-onset cancer survivors exhibit higher late mortality relative to the general population compared to late-onset survivors, with observed disparities varying across sociodemographic subgroups. The association appears largely attributable to malignant neoplasms, though residual confounding and study limitations preclude causal inference. These patterns highlight the need for targeted survivorship monitoring, especially for socioeconomically disadvantaged early-onset survivors.

## Data Availability

Data, protocols, and other details of NHANES are available online at https://wwwn.cdc.gov/nchs/nhanes.
